# How Have Researchers Estimated the Impact of Excess Weight on Mortality? A Systematic Review

**DOI:** 10.1007/s13679-026-00719-2

**Published:** 2026-05-25

**Authors:** Guadalupe García, Cristina Candal-Pedreira, Julia Rey-Brandariz, Lucía de Luis Cid, Mónica Pérez-Ríos

**Affiliations:** 1 Preventive Medicine and Public Health Department, University of Santiago de Compostela (Universidade de Santiago de Compostela), Santiago de Compostela, Spain; 2 Health Research Institute of Santiago de Compostela (Instituto de Investigación Sanitaria de Santiago de Compostela), Santiago de Compostela, Spain; 3 Cross-disciplinary Research in Environmental Technologies (CRETUS), University of Santiago de Compostela, Santiago de Compostela, Spain; 4 Consortium for Biomedical Research in Epidemiology and Public Health, (CIBER en Epidemiología y Salud Pública), Madrid, Spain

**Keywords:** Overweight, Obesity, Body mass index, Mortality

## Abstract

**Objective:**

The aim of this review is to describe and reflect on the methodology of the studies that estimate the attributable mortality (AM) to excess weight. The purpose was to provide a methodological framework and serve as a starting point to improve the estimation of AM to excess weight. This will lead to more comparable estimates over time and across countries.

**Methods:**

A systematic review was conducted considering MEDLINE (Ovid), Embase and Web of Science databases. Studies that had estimated AM to excess weight published until December 2024 were included. Data extraction was performed in an ad-hoc table based on the STREAMS-P tool, and a descriptive analysis of the methodology employed was performed.

**Results:**

The search strategy yielded 2,163 results. Twenty-three studies estimating AM in four continents were included. The majority estimated AM to all-cause mortality (*n* = 9) and for cardiovascular diseases (*n* = 9), followed by cancer without specifying which type of cancer (*n* = 7). Variation was observed in the body mass index (BMI) cut-off points used to define overweight, obesity and excess weight. Thirteen studies applied the population attributable fraction (PAF) formula denoted by PAF = [P (RR-1)] / [1 + P (RR-1)]. Most of the relative risks (RR) derived from meta-analysis (*n* = 14), and only a minority studies (*n* = 6) used RR from national studies based on the population for which AM was estimated.

**Conclusion:**

This review emphasizes methodological constraints on AM estimation to excess weight. Causes of death causally associated with excess weight should be prioritized rather than relying solely on all-cause mortality. The ideal scenario would be to consider lag times; RR derived from the population under study and to use WHO BMI cut-offs and measured data rather than self-reported weight and height. The absence of this data should not prevent AM estimation if limitations are acknowledged. Expanding available estimates worldwide is essential to improve understanding of the mortality burden associated with excess weight.

**Supplementary Information:**

The online version contains supplementary material available at 10.1007/s13679-026-00719-2.

## Introduction

According to the most updated estimates made by the Global Burden of Disease, in 2021, almost half of the adults aged 25 years and older worldwide were living with overweight and obesity. This same study projects a continued increase in this prevalence, with a predicted total of 3.80 billion (95% confidence interval 3.39–4.04) individuals affected on a global scale by 2050 [1].

There is evidence supporting the association between a high body mass index (BMI) and an elevated risk of developing cardiovascular or metabolic diseases, such as hypertension, dyslipidaemia or diabetes mellitus [2, 3]. Also, in 2002, The International Agency for Research on Cancer (IARC) associated excess body weight with different types of cancers such as oesophageal adenocarcinoma, colorectal, liver, renal and postmenopausal breast cancer [4]. After adjusting by smoking status and pre-existence of chronic diseases, overweight and obesity were associated with higher mortality [5].

Estimating attributable mortality (AM) for specific risk factors is a well-established approach to quantify their population-level burden, providing an interpretable measure that raises public awareness and informs policymakers and healthcare professionals in prioritizing prevention and intervention strategies [6]. However, the assessment of overweight and obesity AM has been the subject of debate since the publication of the earliest estimates. This issue has been discussed within the research community, particularly in the United States (U.S.) where data sources and estimation methods have been the subject of debate [7, 8]. The controversy arose after the publication of a set of U.S. AM estimates in which overweight was not associated with increased mortality and even appeared to slightly reduce risk. The authors of these estimates and their methods were closely scrutinized [9]. Nevertheless, since that time, the estimation of AM to overweight and obesity has been established and replicated. On the other hand, it also has been observed that when attributing mortality to other risk factors, a key aspect of the estimation of AM is the underlying data and input parameters used, including its source, definition and comparability. A Spanish simulation study assessed the impact of the exposure definition and operationalization of observed mortality within the estimation framework. The findings showed that the variation in these inputs can result in up to an eightfold difference in AM to second-hand tobacco smoke (SHS) estimates [10].

The aim of this review is to describe and critically reflect on the methodology of studies estimating attributable mortality to excess weight (overweight and obesity), with particular attention to the definition and specification of input data. The purpose was to provide a methodological framework and serve as a starting point to improve the estimation of AM to excess weight. This will lead to more comparable estimates over time and across countries.

## Methods

A systematic review was performed following the PRISMA 2020 (Preferred Reporting Items for Systematics Reviews and Meta-Analyses) guidelines [11]. The review protocol was registered in the PROSPERO Database under the number CRD42025648887.

### Search Strategy

A bibliographic search was performed on December 2024 in MEDLINE (Ovid), Embase and Web of Science databases. The search strategy was designed by a documentalist with expertise in the biomedical area. It combined MeSH, Emtree and free terms. The MeSH and Emtree terms included were: “Mortality”, “Attributed”, “Obesity”, “Overweight” and “Body Mass Index”, among others. No restrictions of country, study period, study design or language were applied. The bibliographic search strategy can be found in Table S1. Furthermore, the references of the included studies were reviewed and considered for inclusion in this review.

### Inclusion/Exclusion Criteria

All studies that estimated AM due to excess weight, following the calculation of the Population Attributable Fraction (PAF), were included. The concept of excess weight was considered as overweight or obesity on its own or the combination of both, or high body mass index (BMI) or excess adiposity. Studies that calculated the Population Attributable Fraction (PAF) but did not estimate the number of attributed deaths were excluded. Simulation studies, studies that did not describe their methodology or provide information on the data sources used were excluded. Additionally, conference abstracts, letters to the editor, editorials and opinion articles were excluded. Studies published in languages different from Spanish, English or Portuguese were excluded.

### Selection of the Studies and Data Extraction

After eliminating duplicates, two researchers screened titles and abstracts following the inclusion/exclusion criteria, through a blinded peer review process. Afterwards, potentially included articles were reviewed full-text following the same review process. Discrepancies were discussed with a third researcher and settled by consensus.

Data extraction was performed in an ad-hoc table based on the STREAMS-P tool, a guideline designed to guide the performance of AM studies to a given risk factor [12] and adapted to the STROBE checklist [13]. This was performed by two researchers, both blindly and independently. Differences of opinion were discussed and settled by consensus, with a third researcher.

For each study, information was extracted regarding (1) publication characteristics (author, journal and year of publication), (2) study characteristics (objective, country and year or period to which estimation refers), (3) population characteristics (age, sex), (4) excess weight definition and (5) methodological characteristics. Point 5 included data on prevalence of excess weight, its source, year of the prevalence, and excess weight category classification, and if the prevalence came from self-reported or measured data; relative risks used and their source; data on PAF and its formulae; observed mortality, its source, year of mortality data and the causes of death assessed, and number and percentage of attributable deaths to excess weight. In instances where the information was differentiated by sex, such data were recorded. In this paper, the focus will be on the characteristics of the methodology used. A descriptive analysis of the studies was performed.

To assess the quality of the studies, the STREAMS-P tool was used [12]. In this tool, quality was assessed by answering yes/no/NS (not specified) to 30 domains. The domains and their analysis can be found in Table S2.

## Results

**Fig. 1:**
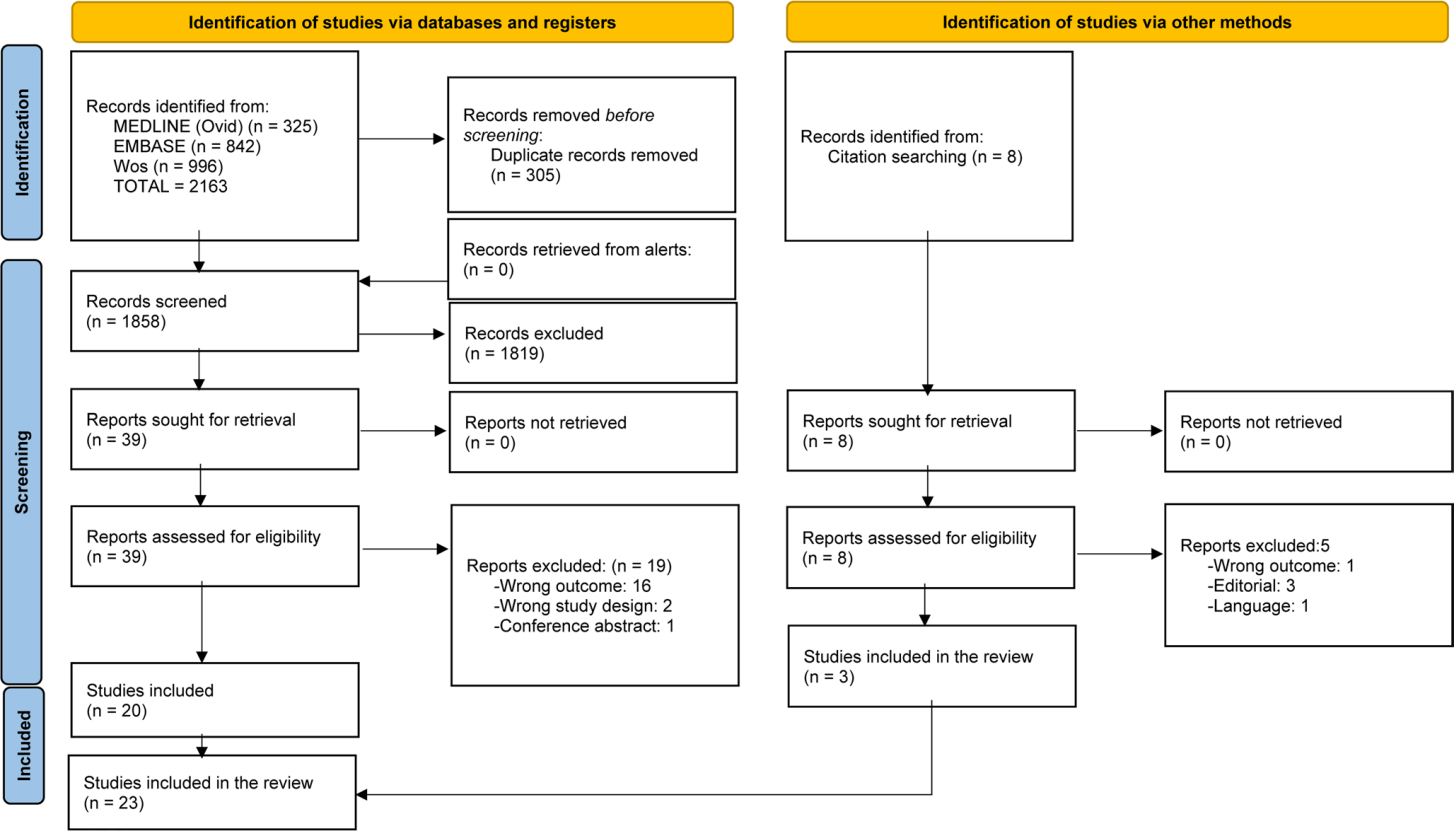
PRISMA flowchart of included studies

The search strategy returned 2,163 results. After removing 305 duplicate references, 1,858 were screened by title and abstract. Of them, 39 were considered for full-text reading. Twenty studies fulfilled the inclusion criteria. Eight references were identified by citation searching, of which three were included. Twenty-three studies were included in this review (Fig. 1).

The included studies were published between 1999 and 2023. The oldest estimation dated from 1985 [14], while the most recent was from 2021 [15]. There were estimations from four continents (Asia, Europe, America and Oceania). The majority (*n* = 5) were performed in the United States (U.S.) [9, 16–19], followed by Canada [14, 20], China [21, 22], and Germany [23, 24] with two estimations for each country. Italy [25], Spain [26], England and Wales [27], The Netherlands [28], Iran [29], New Zealand [30], Mexico [15], Chile [31], Indonesia [32] and Taiwan [33] had one estimation for each country. Two studies estimated AM for the European Union [34, 35] (Table 1).

**Table 1:** General characteristics of the studies

Author	Year of publication	Country	Year/Period of the estimation	Sex	Age
Allison, D. B.	1999	US	1991	Both	≥ 18
Arreola-Ornelas, H.	2023	Mexico	2021	Both	≥ 20
Banegas, J. R.	2003	European Union (Austria, Belgium, Denmark, Finland, France, Germany, Greece, Ireland, Italy, Luxembourg, The Netherlands, Portugal, Spain, Sweden, UK)	1997	Both	≥ 25
Di Maso, M.	2023	Italy	2020	Both	≥ 18y
Djalalinia, S.	2015	Iran	2011	Both	25-65y
Flegal, K. M. − 1	2005	US	2000	Both	≥ 25y
Flegal, K. M. − 2	2007	US	2004	Both	≥ 25y
Islami, F.	2018	US	2014	Both	≥ 30y
Katzmarzyk, P. T.	2004	Canada	1985–2000	Both	20-64y
Kelly, C.	2009	England and Wales	2003	Both	≥ 30y
Konnopka, A.	2011	Germany	2002	Both	≥ 15y
Kristina, S. A.	2021	Indonesia	2011	Both	NS
Lehnert, T.	2015	Germany	2008	Both	≥ 18y
Luo, W.	2007	Canada	2004	Both	≥ 25y
Martín-Ramiro, J. J.	2014	Spain	2006	Both	35-79y
Mehta, N. K.	2009	US	1994	Both	50-61y
Ni Mhurchu, C.	2004	New Zealand	1997	Both	NS
Riquelme, R.	2021	Chile	2018	Both	20-96y
Vidra, N. − 1	2018	The Netherlands	2013	Both	30-75y
Vidra, N. − 2	2018	Europe: Czech Republic, Germany, Finland, France, Hungary, Italy, Poland and the UK	1990–2012	Both	20-79y
Wang, D.	2012	China	2005	Both	NS
Wen, C. P.	2009	Taiwan	2001	Both	≥ 20y
Xu, L. S.	2023	China	2015	Both	NS

*NS* not specified, *UK* United Kingdom, *US* United States of America, *y* years

Nineteen studies estimated AM encompassing both sexes; 10 studies also presented their results differentiated by sex [18, 19, 21–27, 31, 32] and another study presented results exclusively for men [29]. All studies, except one which estimated AM from age 15 [23], considered only the adult population, mostly defined as individuals aged 20 years and older (*n* = 6) [14, 15, 21, 31, 33, 35] (Table 1).

The causes of death considered varied between studies. The majority estimated AM to all-cause mortality (*n* = 9) or exclusively for cardiovascular diseases (*n* = 9). Fourteen studies estimated AM to cancer; seven studies specifying the type of cancer, being the most frequent to be considered the obesity related cancers [19, 21, 22, 25, 26, 30, 32]. Four studies estimated AM considering diabetes mellitus as cause of death. Ten studies specified the ICD (International Classification of Diseases) code (Table 2).

**Table 2:** Methodological characteristics of the studies

Author	Mortality causes considered					Is the ICD code specified?	Excess weight definition	Prevalence source	Data used for estimating prevalence (self-reported data or measured)	RR/HR source	OM source	Year of OM
	All-cause mortality	Cancer	Cardiovascular disease	Diabetes mellitus	Other							
Allison, D. B.	x					No	WHO: Overweight and obesity (2 grades)	NHANES III (1997)	NS	Alameda County Health Study; Tecumseh Community Health Study; Framingham Heart Study; American Cancer Society´s Cancer Prevention Study I (CPS I); Nurse´s Health Study; NHANES I Epidemiologic Follow up Study	MMWR Morb Mort Wkly Rep	1991
Arreola-Ornelas, H.					x	No	GBD: Overweight and obesity	GBD 2021	NS	GBD 2021	NS	NS
Banegas, J. R.	x	x	x			No	WHO: Overweight, obesity and excess weight	Pan-EU survey 1997	self-reported	CPS I (American Cancer Society´s Cancer Prevention Study) for all cause total mortality; Lew and Garfinkel for cancer and cardiovascular mortality	World health statistics Annual	1995–1996
								MONICA, CINDI and Bergstrom et al. 2001	measured	CPS I for smoking nor- or never smokers Allison 1999; CPS I and II for pre-existing illness for healthy never smokers Calle 1999; Troiano 1996 for EU healthy non smokers		
Di Maso, M.		x				Yes	NS	Multiscopo sulle famiglie: aspetti della vita quotidiana, Instituto Nazionale di Statistica 2005	NS	Turati et al.; Xue et al.; Sohn et al.; Alsamarrai et al.; Munsell et al.; Zhang et al.; Kalliala et al.; Liu et al.	National vital statistics on cause of death and GLOBOCAN (for cause specific cancer deaths)	2020
Djalalinia, S.					x	No	Excess body weight > 21 kg/m2	Noncommunicable Disease Surveillance Survey (NCDSS) 2005–2009; 2011	NS	Meta analysis (Danaei G et al. 2014)	Death Registration System DRS collected by Ministry of Health and Medical Education (MOHME)	1996–2010
Flegal, K. M. − 1	x					No	WHO: Overweight and obesity (2 grades)	NHANES 1999–2002	measured	NHANES I (1971–1975), NHANES II (1976–1980) and NHANES III (1988–1994), calculated using Cox proportional hazard models with age as the time scale	US vital statistics data	2000
Flegal, K. M. − 2		x	x		x	Yes	WHO: Overweight and obesity (2 grades)	NHANES 1999–2002	measured	NHANES I (1971–1975), NHANES II (1976–1980) and NHANES III (1988–1994), calculated using Cox proportional hazard models with age as the time scale	US vital statistics data	NS
Islami, F.		x				Yes	WHO: overweight and obesity (3 grades)	NHANES 2011–2014	NS	NS	CDC’S National Center for Health Statistics	NS
Katzmarzyk, P. T.	x					No	WHO: Overweight and obesity (3 grades)	1985 and 1990 Health Promotion Surveys; 1994,1996 and 1998 National Population Surveys; 2000 Canadian Community Health Survey	self-reported	Canada Fitness Survey 1981 (CFS), adjusted for age, gender, cigarette smoking and alcohol consumption, linked with the Canadian Mortality Database	CAN-SIM (national database)	2002
Kelly, C.					x	No	Excess body weight > 21 kg/m2	1997–2004 Health survey for England (HSE)	NS	James et al. 2004	Office for National Statistics	2003
Konnopka, A.		x	x		x	Yes	WHO: Overweight and obesity (3 grades)	German Federal Statistics Office	NS	Calle et al. and Must et al.	German Federal Statistics Office	NS
Kristina, S. A.		x	x	x	x	No	WHO: Overweight and obesity	WHO global health observatory (GHO) database 2010	NS	Meta analysis Guh DP et al. 2009	WHO mortality data	2011
Lehnert, T.		x	x		x	Yes	WHO: Overweight and obesity (3 grades)	For 18-74y: German Health Interview and Examination Survey for Adults (2008); for 75-100y: German Study on Ageing, Cognition, and Dementia in Primary Care Patients (AgeCoDe) 2008	NS	Calle et al. and Must et al.	German Federal Statistics Office	NS
Luo, W.					x	No	WHO: Obesity	2004 Canadian Community Health Survey 2.2	measured	Flegal et al. 2005	Canadian Mortality Database 2002 (considered 2004 by applying the 2002 mortality rate to the 2004 Canadian population)	2004
Martin-Ramiro, J. J.		x	x	x		Yes	WHO: Overweight and obesity	Encuesta Nacional de Salud 2006	self-reported	Whitlock G et al. 2009	Instituto Nacional de Estadistica	2006
Mehta, N. K.	x					No	WHO: Overweight and obesity (3 grades)	Health and Retirement Study 1992–2004 (HRS)	self-reported	Health and Retirement Study (HRS) cohort, calculated from cox models adjusted for sociodemographic variables, socioeconomic status, behaviors (smoking and physical activity)	Health and retirement study	2004
Ni Mhurchu, C.		x	x	x		No	Excess body weight > 21 kg/m2	1997 National Nutrition Survey by age and sex	measured	Cardiovascular diseases (Asia Pacific Cohort Studies Collaboration), Diabetes (published and unpublished sources, James et al.), colorectal cancer and postmenopausal breast cancer (Bergstrom et al.)	New Zealand Health Information Service (NZHIS) mortality database	1997
Riquelme, R.	x	x	x		x	Yes	WHO: Excess weight	National Health Survey of Chile 2016–2017	measured	Global BMI Mortality Collaboration meta-analysis 2016	Official deaths statistics of the Ministry of Health	2018
Vidra, N. − 1	x	x				No	High BMI ≥ 23 kg/m2; Obesity BMI ≥ 30 kg/m2	Dutch Health Interview Survey from Statistics Netherlands 1981–2013	self-reported	Flegal et al. 2013; Wang 2015; Lobstein and Leach 2010; Daneani et al. 2009	All cause: Human Mortality Database (HDM)	1981–2013
											Cause specific: World Health Organization Mortality Database	NS
Vidra, N. − 2	x					No	WHO: Obesity	Global Burden of Disease Ng et al. 2014	NS	Flegal et al. 2013 (adjusted for age, sex, and smoking)	Human mortality database	2016
Wang, D.		x				Yes	WHO: Overweight, obesity and excess weight	1992 China National Nutrition Survey	NS	Korean Cancer prevention study 2008; calculated based on Shangai Women’s Health Study; Korean Cancer prevention study 2008	The Third National Death Cause Survey	2005
Wen, C. P.	x	x	x	x		Yes	WHO Western Pacific Regional Office: obesity (≥ 25 kg/m2)	National Health Interview Survey (NHIS) 2001, cohort 1989–1992 of > 40 years	self-reported	A cohort (cited three articles: Ho MS 1993; Wen et al. 2004; Wen et al. 2005). Calculated by the study and adjusted for age, sex, smoking, alcohol drinking and physical activity	National mortality data	2001
Xu, L. S.		x				Yes	WHO: overweight, obesity and excess weight	China Health and Nutrition Survey (CHNS) 2004	NS	Meta analysis - Schmid et al. 2015; Niedermaier et al. 2015; Zhang et al. 2014; Jiao et al. 2010; Yang et al. 2020; Tan et al. 2015; Yang et al. 2009/Cohort - Chen et al. 2010; Nam et al. 2019/Pooled analysis - Birmann et al. 2018; Parr et al. 2010	Chinese Cancer Registry Annual Report	2009,2013 and 2018

*BMI* Body mass index, *HR* hazard ratio, *NS* not specified, *OM* observed mortality, *RR* relative risk, *WHO* World Health Organization

Regarding the definition of excess weight, 17 studies used the World Health Organization (WHO) BMI cut-off points. However, these studies differed in the categories considered: some included overweight or obesity on their own; others only obesity (≥ 30 kg/m^2^), some distinguished between obesity grades I, II and III, and also one study considered excess weight (≥ 25 kg/m^2^), combining overweight and obesity (Table 2) [31]. Another study from Taiwan considered WHO Western Pacific Regional Office cut-off points on which obesity is classified as ≥ 25 kg/m^2^ [33]. Regarding the remaining six studies: three considered excess body weight as > 21 kg/m^2^ [27, 29, 30], one considered a Global Burden of Disease (GBD) overweight and obesity definition [15]; one considered high BMI as BMI ≥ 23 kg/m^2^ and obesity as BMI ≥ 30 kg/m^2^ [28]; and the remaining study did not specified the excess weight definition [25].

Of the 23 studies, 20 specified the formula used to calculate PAF, including 13 that applied the standard formula, PAF = [P (RR − 1)]/[1 + P (RR − 1)], and one that compared three alternative formulas [28]; the remaining three studies did not report the formula [9, 15, 17]. With respect to sex and age group, eleven studies calculated PAF by sex [19, 22–26, 29, 31, 32, 35, 36], and three by age groups and summed the resulting AM to obtain overall AM [23, 24, 29] (Table 3).

**Table 3:** Population attributable fraction methods

PAF Method	Authors applying the method
PAF = ∑ [P (RR-1)/RR]	Allison et al.; Katzmarzyk et al.
PAF= [P (RR −1)]/[P (RR-1) + 1]	Banegas et al.; Di Maso et al.; Konnopka et al.; Lehnert et al.; Kristina et al.; Luo et al.; Martín Ramiro et al.; Vidra et al.; Wang et al.; Wen et al.; Xu et al.; Islami et al.
Comparative risk assessment (CRA) PAF = (Factual population risk - Counterfactual population risk)/Factual population risk// PAF = (∑P1 RRi − 1)/∑P1 Rri	Djalalinia et al.; Riquelme et al.; Vidra et al.; Kelly et al.; Ni Mhurchu et al.
Counterfactual method (NS)	Flegal et al.

All the studies specified the source of the prevalence of excess weight, with 11 indicating whether the data were self-reported [14, 18, 26, 28, 33] or measured [9, 17, 20, 30, 31]. One study compared estimates from both self-reported and measured data (Table 2) [34].

Except for one study [19], all specified the relative risks (RR) used to calculate the PAF (Table 2). In 14 studies the RR were obtained from meta-analysis, where in four were based on populations that corresponded to those to which the AM was estimated [22, 28, 33, 34]. Of the remaining eight studies, one used RR reported by GBD [15] and seven relied on RR that correspond to studies conducted at the national level, six of which matched the target population [9, 14, 16–18, 21], while one applied U.S.-based RR for all-cause mortality and RR from meta-analysis for cancer and cardiovascular mortality to the European population [34]. Six studies specified using adjusted RR by several variables such as age, sex/gender, or tobacco and alcohol consumption [14, 16, 18, 33–35] (Table S3).

In two studies [15, 18], no information on observed mortality was provided; while in the remaining studies, 15 specified the year to which the mortality data referred. In seven of these studies, the year of the observed mortality data was later than that of the prevalence [14, 21, 22, 25, 29, 31, 32, 35], with three studies considered a lag time between 10 and 15 years [21, 22, 25], and the remaining one to two years (Table 2).

Regarding the quality assessed by the STREAMS-P tool, the main deviations arose from the domains “Prevalence respect age-dependent categories of exposure”, and “Prevalence respects disease-specific latency time”. The domains with less deviations were “Causes included are causally related with the risk factor under study” and “Mortality data are registry-based” (Table S2).

## Discussion

This review shows that the estimation of AM due to excess weight is methodologically inconsistent and remains confined to a limited set of countries (United States, Canada, Mexico, Chile, Germany, Italy, Spain, England and Wales, The Netherlands, China, Iran, Indonesia, Taiwan, New Zealand, and the European Union as a whole). Studies spanned to 14 countries and covered nearly all continents except Africa. However, most studies originated from the U.S., which had five estimates. It should also be highlighted that the estimates covered only 7% of all countries. This underscores a gap in global evidence. Estimates are available since 1985, with the first publication dating from 1999 [16], showing that the characterization of the obesity epidemic and its burden has been of interest for a considerable period. Despite this, given the importance of this quantification, relatively few studies have been performed, as evidenced by the 23 studies included in this review. This is further supported by comparisons with systematic reviews that summarize AM estimates for other risk factors, such as tobacco, where the number of included articles exceeds 50 [37].

No clear pattern was observed in the methodology used to estimate AM to excess weight. This aspect creates challenges in comparing the impact of excess weight on mortality across populations, countries and over the time. Nevertheless, methodological differences in estimating AM are not exclusive to excess weight. Previous reviews have also shown that such differences appear when estimating AM related to tobacco consumption and SHS [37, 38]. In fact, some of the methodological inconsistencies identified in this review, such as the variation on causes of death and the relative risks (RR) used, are consistent with those reported in these reviews.

Overall, the main methodological constraints were that studies focused on applying prevalence data contemporaneous with mortality (20 studies), estimating excess weight AM referring to all-cause mortality (nine studies), and risk estimates derived from meta-analysis (14 studies). In most cases, (ten studies) the RR used were not related to the population under study.

### Absence of a Lag Time Considered

When evaluating the methodology of the studies included in this review, a key aspect to consider is the failure to account for a lag time between the exposure (excess weight) and the outcome (mortality) in the estimation of AM. In most cases, mortality data are contemporaneous with prevalence data, without incorporating a temporal delay between exposure and outcome. Only three studies considered a latency period of 10 to 15 years [21, 22, 25], all of which estimated the impact of excess weight specifically on cancer-related mortality. Conversely, several authors have hypothesized the existence of a specific lag period required for the development of cardiovascular diseases in individuals with obesity [39, 40]. For instance, a 10 years lapse is proposed for hypertension [41]. In this review, nine studies assessed AM related to cardiovascular diseases. In those studies, mortality may have been overestimated because they did not include a latency period. In general, the lack of consideration of lag times should be noted, since mortality could be overestimated in countries where obesity prevalence is increasing.

### Differences in Causes of Death Considered

In this review, the most common approach to estimate AM due to excess weight was based on all-cause mortality analysis. All-cause mortality includes deaths from, for example, infectious diseases that are not related to obesity. This approach may overestimate AM, as it includes deaths not causally associated to excess weight [12]. Nevertheless, all-cause mortality may be the only feasible option in countries with low-quality mortality records. It has been estimated that in 2019, only 54 countries worldwide had complete mortality records and high-quality cause of death registration, meaning that only 23% of deaths globally were comprehensively recorded. Furthermore, there were significant differences depending on the region. While in the Americas and Europe more than 90% of deaths were recorded with their causes, in the Eastern Mediterranean and Africa these percentages dropped to 27% and 8%, respectively [42]. In relation to causes of death associated with excess weight, the IARC reported in 2002 the types of cancer associated with overweight and obesity, ruling out those with no association [4]. These results were confirmed in the 2018 IARC Handbook (Volume 16) [43]. Incorporating this evidence is essential for obtaining valid estimates of AM to excess weight. In this review, 14 studies estimated AM to cancer, seven of which made the estimation for obesity-related cancers.

The association between diabetes and obesity is well-established; however, the inclusion of diabetes mellitus as a cause of death in AM estimates was considered in only four studies [26, 30, 32, 33]. The exclusion of diabetes from the AM estimation leads to an underestimation of the mortality burden. It is important to highlight that physicians tend to omit diabetes on death certificates if unaware of a prior diagnosis, potentially leading to underestimation of AM to excess weight. For example, in one study, diabetes was recorded as the underlying cause of death for only 10% of people living with diabetes and was referenced elsewhere on the certificate for 39% [44].

### Selection of the Relative Risks and Possible Confounders

In the AM analysis included in this review, the use of RR that correspond to the population under study was limited. Most studies instead relied on meta-analysis combining cohort and case-control data from multiple countries. According to Flegal et al., the utilization of these RR may result in the presence of bias, given that they do not precisely replicate the risks that would be observed in the population under study [45]. Even so, only a few of the included studies explicitly identify this as a limitation [23, 24, 28]. Availability of country-specific mortality risks associated with excess weight remains a challenge, as long-term cohort or large case-control studies are not always available in most countries. This explains the use of RR from meta-analysis in these cases, as it can provide precise estimates if all available evidence is compiled. Indeed, the estimation of AM to tobacco consumption often relies on meta-analysis-derived RR, even when cohort-derived data are available [12, 46]. In the case of applying risks derived from meta-analysis, it would be advisable to consider the populations characteristics and, to meta-analyze the results separately by geographical regions with similar trends in the obesity epidemic.

A further consideration regarding RR, is confounder adjustment, which varies across the scientific literature. A potential confounder is the presence of pre-existing chronic diseases at baseline, which may contribute to unintentional weight loss and increase the risk of mortality [34, 47]. To address this potential bias, some authors have proposed excluding individuals living with such conditions at study inception [48]. However, it has also been acknowledged that, given the complex interplay between weight status, disease and mortality, the possibility of residual confounding cannot be ruled out [18]. Smoking represents a debated confounding factor in the estimation of AM related to excess weight. The considerable independent mortality risk conferred by tobacco consumption complicates the accurate quantification of the mortality burden attributable to excess weight [49]. Among the studies included in this review, eight explicitly reported using RR adjusted for smoking status [14, 16, 18, 28, 33–35]. Furthermore, some authors have observed that the risk of death associated with obesity in smokers is lower than in non-smokers [20, 50]. This may be partly explained by the fact that smokers tend to increase their energy expenditure by approximately 10%, resulting in weight loss and a lower BMI [20, 50]. Indeed, a review suggested that, in the U.S., smokers weigh on average 4 to 5 kg less than non-smokers [50]. These differences have important implications for the estimation of the prevalence and risks, impacting the PAF. For instance, Stokes et al. reported a PAF of 31.9% attributable to high BMI among never-smokers in a U.S. cohort. In contrast, the proportion was 11.3% among current smokers. The authors concluded that the lower RR observed in smokers largely explained the substantially reduced PAF in this group. This finding was also attributed to the dominant risk posed by tobacco consumption itself, which independently accounts for a substantial number of deaths [49].

Physical activity is another relevant confounder, as active individuals are likely to have lower BMI and less adiposity [51]. Other confounders not commonly considered may exist, including recent unintentional or long-term intentional weight loss and duration of overweight or obesity [34].

### Importance of the BMI Cut-Off Points and Source of Data to Estimate Prevalence

An important methodological issue that affects the comparability of results across studies is the variation in the BMI cut-off points used to estimate the prevalence of overweight and obesity. While some studies report AM estimates separately for overweight and obesity [22], others combine both categories [27, 30, 31], and another’s considers only obesity [20, 33, 35], with further differences in the obesity grades [9, 14, 16, 17, 19, 24]. These inconsistencies complicate the interpretation and comparison of findings across the existing literature. Another relevant aspect is debated principally among the Asian studies [33]. Evidence indicates that the average BMI in South and East Asian countries is generally lower than that observed in North America and Europe [52]. Furthermore, various studies indicate an increased risk of metabolic diseases, such as diabetes, from a BMI of 23 kg/m^2^, as well as an increased risk of mortality from cardiovascular diseases from 25 kg/m^2^ in Asian populations [53, 54]. This suggests that Asian populations may have an increased risk of diabetes and cardiovascular diseases at lower BMI levels than non-Asian populations. This increased risk is hypothesized to be related to a higher percentage of body fat at lower BMI values. In addition, differences in BMI across Asian ethnic populations have been documented. For example, Chinese and rural Thai women have values similar to Europeans, whereas this is not observed in other Asian women [52, 55]. These differences suggest that the use of the general cut-off points proposed by the WHO for the population of the Asian region can be inaccurate [53]. Nevertheless, a WHO expert consultation group concluded that the current BMI cut-off point of 25 kg/m² does not provide an adequate basis for assessing risks related to overweight and obesity in many Asian countries, in addition to recognizing the aforementioned differences. Further studies are needed to establish appropriate cut-off points for this population [55].

A critical factor in estimating AM due to excess weight is the source of anthropometric data, as self-reported measures tend to underestimate overweight by 1.8–3.9 and obesity by 0.7–13.4% points [56]. In this review, only half of the studies specified their data source, evenly split between self-reported and measured data. Biases in self-reporting, particularly overreported height and underreported weight among women, lead to underestimated overweight and obesity prevalence and likely an underestimation of the mortality burden attributable to excess weight [56–58]. Nevertheless, two issues could be considered: (1) in epidemiology, the use of self-reported weight and height is common, a method both simpler and less expensive than taking objective data. Given these limitations, countries commonly rely on self-reported nationally representative data on weight and height to estimate the prevalence of overweight and obesity. Although measured data would be ideal, this is not commonly available. Self-reported data therefore represents a necessary and appropriate alternative in the absence of measured data. (2) When self-reported data are the only available option, the associated error and bias may be systematic and consistent over time. This consistency may help preserve the validity of temporal comparisons. Undoubtedly, this aspect is relevant when estimating AM to excess weight, since one of the studies included compared the results obtained from self-reported and measured data from European Union countries [34]. This study concluded that self-reported data underestimated the true prevalence of obesity, as it was about 6% lower than when using measured data. Estimates based on self-reported data provide a conservative estimate of the impact of excess weight on population mortality. Nevertheless, it should be noted that the study specified that the measured data was not representative of the EU countries.

### Limitations and Strengths

In this review, the bibliographic search was limited to biomedical databases, which may have excluded relevant data not indexed in these sources. However, multiple databases were consulted, resulting in the identification of a substantial number of studies, thereby minimizing the likelihood of missing pertinent information. In addition, the reference lists of the included studies were screened to identify potentially relevant studies not captured by the initial search. Furthermore, no language restrictions were applied to the search strategy, and studies published in English, Spanish and Portuguese were also considered for inclusion in this review.

The quality of the studies was evaluated using STREAMS-P, a validated tool specifically designed to assess AM studies. Although this tool does not provide a global quality score for each study, this should not be considered a limitation. On the contrary, in the context of scrutinizing the methodology of AM studies, this represents a strength. It allows for the identification and detailed assessment of each domain by addressing the key components relevant to estimating AM.

A major strength is that a thorough peer review of the studies was conducted independently, with the involvement of researchers who have expertise in the field of AM to different risk factors. This approach also facilitated the methodological evaluation of the studies.

### Conclusion

The estimation of attributable mortality constitutes a highly pertinent measure for decision-making and raising awareness regarding prevalent risk factors such as overweight and obesity. When it comes to overweight and obesity, estimations remain restricted to a limited number of countries. This review highlights important methodological considerations that should be taken into account and may serve as a starting point for the scientific community and decision-makers to reach a consensus on these issues.

In our view, the selection of causes of death should, whenever possible, be those with evidence of association with excess weight, such as obesity-related cancers, cardiovascular diseases, and metabolic disorders, while avoiding the use of an exclusively all-cause mortality approach when feasible. This last approach might overestimate the actual burden of disease. Lag times should be considered as mortality could be overestimated, especially in regions where obesity prevalence is increasing. The relative risks used should be derived from the population under study. The WHO BMI cut-off points should be used to permit comparability, including the full categorization provided by this organization (overweight, obesity, and its grades). Finally, measured data should be favoured over self-reported data. Likewise, by standardizing these factors, future studies can minimize bias, enhance comparability, and provide more accurate assessments of the mortality burden attributable to excess weight across diverse populations. Nevertheless, these represent ideal conditions for the estimation of attributable mortality. When appropriately acknowledged, the absence of such data should not be considered a barrier to estimating attributable mortality.

Given that attribution studies are not commonly performed and only a small proportion of countries currently have available estimates, this lack represents an important gap in knowledge. Increasing the number of estimates available worldwide, ideally comparable, would improve knowledge of the burden of overweight and obesity.

## Key References

- GBD 2021 Adult BMI Collaborators. Global, regional, and national prevalence of adult overweight and obesity, 1990–2021, with forecasts to 2050: a forecasting study for the Global Burden of Disease Study 2021. Lancet [Internet] 2025 Mar 8;405(10481):813 − 38. doi: 10.1016/s0140-6736(25)00355-1.
  - *This paper is based on the most recent published study using data from the Global Burden of Disease Study 2021*,* which included more than 1350 information sources and 204 countries. Adults overweight and obesity have risen sharply worldwide since 1990 and are projected to continue increasing through 2050 across most countries. If no effective interventions are implemented more than 50% of the global adult population (approximately 3.8 billion people) will be overweight or obese by 2050.*
- Lim Y BJ. Obesity and Comorbid Conditions. StatPearls, editor. Treasure Island (FL): StatPearls Publishing; 2025.
  - *This article provides a comprehensive clinical overview of obesity*,* including its definition*,* diagnostic criteria*,* and underlying physiological mechanisms. An overview of the epidemiology of obesity and related comorbid conditions and their impact on population health is well described. Additionally*,* it outlines evidence-based approaches for the prevention and management of obesity*,* emphasizing the importance of multidisciplinary care and interprofessional collaboration in clinical practice.*
- Pérez-Ríos M, Rey-Brandariz J, Galán I, et al. Methodological guidelines for the estimation of attributable mortality using a prevalence-based method: the STREAMS-P tool. J Clin Epidemiol [Internet] 2022 Jul;147:101 − 10. doi: 10.1016/j.jclinepi.2022.03.016 PubMed PMID: 35341948.
  - *This article by Pérez-Ríos et al. presents the STREAMS-P tool*,* a methodological framework designed to standardize and improve the estimation of attributable mortality using prevalence-based approaches. It provides structured guidelines and checklists to support both the design and reporting of studies*,* as well as to critically assess their methodological quality. To our knowledge*,* STREAMS-P is the first set of criteria specifically created to assess the quality of such studies.*
- Safizadeh F, Mandic M, Hoffmeister M, Brenner H. Reevaluating the fraction of cancer attributable to excess weight: overcoming the hidden impact of prediagnostic weight loss. European Journal of Epidemiology [Internet] 2024 2024/09/01;39(9):991–1003. doi: 10.1007/s10654-024-01146-0.
  - *The study by Safizadeh et al. evaluates the association between excess body weight (overweight and obesity) and cancer risk*,* specifically obesity related cancers*,* and the importance of pre-diagnostic weight loss. The authors reassess previously reported estimates of the fraction of cancer attributable to overweight and obesity*,* highlighting that weight loss prior to diagnosis may lead to underestimation of the true association.*
- Lynggaard V, Soendergaard SM, Boettcher M, Stampe L, Kempel MK, Winding TN. Bias in self-reported height and weight in a cohort of young adults – perspectives for cardio-metabolic risk prevention programmes. European Heart Journal [Internet] 2022;43(Supplement_2) doi: 10.1093/eurheartj/ehac544.2390.
  - The study by Lynggaard et al. investigates the accuracy of self-reported height and weight in a cohort of young adults by comparing self-reported and objectively measured anthropometric data. It examines systematic bias in reporting and explores how these discrepancies may vary according to sociodemographic and body composition factors.

## Supplementary Information

Below is the link to the electronic supplementary material.

Supplementary File 1 (DOCX 52.9 KB)

## Data Availability

Data for this study are publicly available.

## Declarations

### Statement on the use of artificial intelligence

No artificial intelligence was used.

## Glossary

AM: Attributable Mortality.
BMI: Body Mass Index.
GBD: Global Burden of Disease.
IARC: International Agency for Research on Cancer.
RR: Relative Risks.
PAF: Population Attributable Fraction.
PRISMA: Preferred Reporting Items for Systematics Reviews and Meta-Analyses.
WHO: World Health Organization.
