# Supplementary material for: How Have Researchers Estimated the Impact of Excess Weight on Mortality? A Systematic Review
Source: Curr Obes Rep. 2026 May 25;15(1):41. doi: 10.1007/s13679-026-00719-2 (PMC13201345; doi:10.1007/s13679-026-00719-2)
Supplement: Supplementary file 1 — (DOCX 52.9 KB) [file 13679_2026_719_MOESM1_ESM.docx]

**Table S1: Search strategy**

| **Database** | **Strategy** |
| --- | --- |
| **Embase, MEDLINE (Ovid)** | \| *obesity/ \| \| --- \| \| *Overweight/ \| \| *body mass/ or *body mass index/ \| \| 1 or 2 or 3 \| \| *mortality/ or mortality.sh. \| \| *death/ \| \| 5 or 6 \| \| 4 and 7 \| \| *Overweight/ep, mo [Epidemiology, Mortality] \| \| *Obesity/ep, mo [Epidemiology, Mortality] \| \| 9 or 10 \| \| 11 use medall \| \| 8 or 12 \| \| (((mortality or deaths or death) adj2 (attributed or attributable or associated or risk)) and (overweight or "excess body weight" or "excess weight" or obesity)).ti,ab,kw. \| \| 13 and 14 \| \| limit 15 to (conference abstract or conference paper or "conference review" or editorial or letter or note) \| \| 15 not 16 \| \| 17 use oemezd \| \| limit 15 to (clinical conference or comment or congress or consensus development conference or consensus development conference, nih or editorial or lecture or letter) \| \| 15 not 19 \| \| 20 use medall \| \| 18 or 21 \| \| limit 22 to human \| \| remove duplicates from 23 \| |
| **Web of Science** | TS= (((mortality or deaths or death) NEAR/2 (attributed or attributable or associated or risk)) NEAR/3 (overweight or "excess body weight" or "excess weight" or obesity)) and Letter or Editorial Material or Meeting Abstract or Proceeding Paper or Book Chapters (Exclude – Document Types) |

**Table S2: Quality assessment with the STREAMS-P tool**

| **Author** | **Observed mortality** | | | | | **Prevalence** | | | | | | **Risk** | | | | | | **Results** | | | | | | | | **Discussion** | | | | |
| --- | --- | --- | --- | --- | --- | --- | --- | --- | --- | --- | --- | --- | --- | --- | --- | --- | --- | --- | --- | --- | --- | --- | --- | --- | --- | --- | --- | --- | --- | --- |
|  | **O1** | **O2** | **O3** | **O4** | **O5** | **P1** | **P2** | **P3** | **P4** | **P5** | **P6** | **R1** | **R2** | **R3** | **R4** | **R5** | **R6** | **RE1** | **RE2** | **RE3** | **RE4** | **RE5** | **RE6** | **RE7** | **RE8** | **D1** | **D2** | **D3** | **D4** | **D5** |
| Allison, D. B. | No | Yes | No | Yes | NS | Yes | Yes | No | NS | No | No | Yes | No | No | No | No | No | No | No | No | No | No | No | No | Yes | Yes | Yes | Yes | Yes | No |
| Arreola-Ornelas, H. | No | No | NS | NS | NS | Yes | No | No | NS | NS | No | No | No | Yes | No | No | No | No | No | No | No | No | Yes | No | No | No | No | No | Yes | No |
| Banegas, J. R. | Yes | Yes | No | Yes | NS | Yes | Yes | No | Yes | No | No | Yes | Yes | Yes | No | No | No | Yes | No | No | Yes | No | No | No | No | Yes | Yes | No | Yes | No |
| Di Maso, M. | Yes | No | NS | Yes | NS | Yes | Yes | Yes | NS | Yes | Yes | No | No | Yes | No | No | No | Yes | Yes | Yes | Yes | No | Yes | No | No | Yes | Yes | No | No | Yes |
| Djalalinia, S. | Yes | No | No | Yes | NS | No | No | No | NS | No | No | No | No | Yes | No | No | No | Yes | Yes | No | No | No | No | No | No | No | Yes | No | Yes | No |
| Flegal, K. M. - 1 | No | Yes | Yes | Yes | NS | Yes | Yes | Yes | Yes | No | No | Yes | No | No | Yes | Yes | No | Yes | No | No | Yes | No | No | No | No | Yes | Yes | No | Yes | Yes |
| Flegal, K. M. - 2 | Yes | Yes | Yes | Yes | NS | Yes | No | Yes | Yes | NS | No | Yes | No | No | Yes | Yes | No | Yes | Yes | Yes | No | No | No | No | Yes | Yes | No | No | Yes | Yes |
| Islami, F | Yes | No | No | Yes | NS | Yes | Yes | No | NS | No | No | No | Yes | No | No | No | No | Yes | Yes | No | Yes | No | No | No | No | Yes | Yes | No | Yes | Yes |
| Katzmarzyk, P. T. | No | Yes | No | Yes | NS | Yes | Yes | No | No | No | No | Yes | No | No | No | No | No | No | No | No | Yes | No | No | No | Yes | Yes | No | No | Yes | Yes |
| Kelly, C. | Yes | No | NS | Yes | NS | Yes | No | No | NS | No | NS | No | No | Yes | No | No | No | Yes | No | No | No | No | No | No | Yes | No | Yes | No | Yes | No |
| Konnopka, A. | Yes | No | Yes | Yes | NS | Yes | No | Yes | NS | NS | NS | No | No | Yes | No | No | No | Yes | Yes | No | No | No | Yes | No | Yes | No | Yes | Yes | No | Yes |
| Kristina, S. A. | Yes | No | NS | Yes | NS | NS | Yes | No | NS | No | NS | No | No | Yes | No | No | No | Yes | Yes | No | No | No | Yes | No | No | Yes | No | Yes | Yes | Yes |
| Lehnert, T. | Yes | No | NS | Yes | NS | Yes | No | No | NS | No | NS | No | No | Yes | No | No | No | Yes | Yes | No | No | No | Yes | No | Yes | Yes | Yes | No | No | Yes |
| Luo, W. | Yes | No | Yes | Yes | NS | Yes | Yes | Yes | Yes | No | Yes | No | No | Yes | No | No | No | Yes | Yes | No | Yes | No | No | No | No | Yes | Yes | No | Yes | Yes |
| Martin-Ramiro, J. J. | Yes | No | Yes | Yes | NS | Yes | Yes | Yes | No | No | No | No | No | Yes | No | No | No | Yes | Yes | No | Yes | No | No | No | No | Yes | No | No | Yes | Yes |
| Mehta, N. K. | No | Yes | Yes | NS | NS | Yes | Yes | No | No | Yes | No | Yes | No | No | No | No | No | Yes | No | No | Yes | No | No | No | Yes | Yes | No | Yes | Yes | Yes |
| Ni Mhurchu,C. | Yes | No | No | Yes | NS | Yes | No | No | Yes | No | NS | No | No | Yes | No | No | No | Yes | No | No | No | No | No | No | No | No | Yes | No | Yes | No |
| Riquelme, R. | Yes | Yes | No | Yes | NS | Yes | No | No | Yes | No | Yes | No | No | Yes | No | No | No | Yes | Yes | No | Yes | No | No | No | No | Yes | Yes | No | Yes | No |
| Vidra, N. - 1 | Yes | Yes | Yes | Yes | NS | Yes | No | No | No | No | NS | No | No | Yes | Yes | No | No | No | Yes | No | No | No | No | No | No | No | Yes | No | No | Yes |
| Vidra, N. - 2 | No | Yes | Yes | Yes | NS | No | No | No | NS | No | NS | No | No | Yes | No | No | No | No | No | No | No | No | No | No | Yes | No | No | No | No | No |
| Wang, D. | Yes | No | Yes | Yes | NS | Yes | Yes | No | NS | Yes | No | No | Yes | No | No | No | No | Yes | Yes | Yes | Yes | No | Yes | No | No | No | Yes | No | No | No |
| Wen, C. P. | Yes | Yes | No | Yes | NS | Yes | Yes | No | No | No | No | No | Yes | No | No | No | No | Yes | Yes | No | Yes | No | No | No | No | Yes | Yes | No | Yes | Yes |
| Xu, L. S. | Yes | No | Yes | Yes | NS | Yes | Yes | No | NS | Yes | No | No | No | Yes | No | No | No | Yes | Yes | Yes | Yes | No | Yes | No | Yes | Yes | Yes | No | No | No |

Abbreviation: NS: not specified

Appendix of Table S2: Domain explanation of table S2

| **Code** | **Domain explanation** |
| --- | --- |
| O1 | Causes included are causally related with the risk factor under study |
| O2 | All-cause mortality (when recommended) |
| O3 | Mortality ages under study respect the exposure induction time |
| O4 | Mortality data are registry-based |
| O5 | Proportion of deaths in the category known as "garbage" codes is less than 10% in the studied year/s |
| P1 | Prevalence is representative of the population under study |
| P2 | Prevalence reflects the different categories of exposure |
| P3 | Prevalence respects age-dependent categories of exposure |
| P4 | Prevalence derives from objective measures |
| P5 | Prevalence respects disease-specific latency time |
| P6 | Prevalence estimations are precise (e.g., narrow 95% confidence intervals) |
| R1 | Risk data derived from studies developed in the study region |
| R2 | Risk derived from other regions with a similar epidemiological situation in the risk factor under study |
| R3 | Risk derived from strongly established meta-analyses |
| R4 | Apply age-group specific risks (if necessary) |
| R5 | Age-groups in risk and prevalence are matched |
| R6 | Model synergy of effect modification (if present) |
| RE1 | Report attributed mortality figures in selected groupings |
| RE2 | Report population attributable fractions in selected groupings |
| RE3 | Report observed mortality figures in selected groupings |
| RE4 | Report prevalence figures in selected groupings |
| RE5 | Attributable and preventable deaths are differentiated (if necessary) |
| RE6 | Report additional items (potential years of life lost.) |
| RE7 | Report third-party effects (if necessary) |
| RE8 | Perform a sensitivity analysis |
| D1 | Include a statement on prevalence employed |
| D2 | Include a statement on the risks employed |
| D3 | Include a statement on the observed mortality employed |
| D4 | Report the history of the risk factor on the population under study |
| D5 | Report a statement on the strength of evidence regarding the exposure-risk association |

**Table S3: Source of the relative risk (RR) from published articles**

| **Study** | **RR source - reference** | **Type of study** |
| --- | --- | --- |
| Di Maso, M. | Turati F, Tramacere I, La Vecchia C, Negri E. A meta-analysis of body mass index and esophageal and gastric cardia adenocarcinoma. Ann Oncol. 2013 Mar;24(3):609-17. doi: 10.1093/annonc/mds244. Epub 2012 Aug 16. PMID: 22898040. | meta-analysis  of case-control and cohort studies |
|  | Xue K, Li FF, Chen YW, Zhou YH, He J. Body mass index and the risk of cancer in women compared with men: a meta-analysis of prospective cohort studies. Eur J Cancer Prev. 2017 Jan;26(1):94-105. doi: 10.1097/CEJ.0000000000000231. PMID: 27662398. | metaanalysis  of cohort studies |
|  | Sohn W, Lee HW, Lee S, Lim JH, Lee MW, Park CH, Yoon SK. Obesity and the risk of primary liver cancer: A systematic review and meta-analysis. Clin Mol Hepatol. 2021 Jan;27(1):157-174. doi: 10.3350/cmh.2020.0176. Epub 2020 Nov 26. PMID: 33238333; PMCID: PMC7820201. | metaanalysis  of cohort studies |
|  | Alsamarrai A, Das SL, Windsor JA, Petrov MS. Factors that affect risk for pancreatic disease in the general population: a systematic review and meta-analysis of prospective cohort studies. Clin Gastroenterol Hepatol. 2014 Oct;12(10):1635-44.e5; quiz e103. doi: 10.1016/j.cgh.2014.01.038. Epub 2014 Feb 5. PMID: 24509242. | Metaanalysis  of cohort studies |
|  | Munsell MF, Sprague BL, Berry DA, Chisholm G, Trentham-Dietz A. Body mass index and breast cancer risk according to postmenopausal estrogen-progestin use and hormone receptor status. Epidemiol Rev. 2014;36(1):114-36. doi: 10.1093/epirev/mxt010. PMID: 24375928; PMCID: PMC3873844. | metaanalysis of case control studies, cohort studies, case-cohort studies and clinical trials |
|  | Zhang Y, Liu H, Yang S, Zhang J, Qian L, Chen X. Overweight, obesity and endometrial cancer risk: results from a systematic review and meta-analysis. Int J Biol Markers. 2014 Mar 24;29(1):e21-9. doi: 10.5301/jbm.5000047. PMID: 24170556. | meta-analysis of case-control and cohort studies |
|  | Kalliala I, Markozannes G, Gunter MJ, Paraskevaidis E, Gabra H, Mitra A, Terzidou V, Bennett P, Martin-Hirsch P, Tsilidis KK, Kyrgiou M. Obesity and gynaecological and obstetric conditions: umbrella review of the literature. BMJ. 2017 Oct 26;359:j4511. doi: 10.1136/bmj.j4511. PMID: 29074629; PMCID: PMC5656976. | umbrella review  of metaanalysis |
|  | Liu X, Sun Q, Hou H, Zhu K, Wang Q, Liu H, Zhang Q, Ji L, Li D. The association between BMI and kidney cancer risk: An updated dose-response meta-analysis in accordance with PRISMA guideline. Medicine (Baltimore). 2018 Nov;97(44):e12860. doi: 10.1097/MD.0000000000012860. PMID: 30383638; PMCID: PMC6221676. | metaanalysis of  cohort studies |
| Djalalinia, S. | Global Burden of Metabolic Risk Factors for Chronic Diseases Collaboration. Cardiovascular disease, chronic kidney disease, and diabetes mortality burden of cardiometabolic risk factors from 1980 to 2010: a comparative risk assessment. Lancet Diabetes Endocrinol. 2014 Aug;2(8):634-47. doi: 10.1016/S2213-8587(14)70102-0. Epub 2014 May 16. PMID: 24842598; PMCID: PMC4572741. | metaanalysis  of prospective  studies |
| Kelly, C. | James, W.P.T., Jackson-Leach, R. and NiMhurchu, C. (2004) Overweight and obesity (high body mass index). In: Ezzati, M., Lopez, A., Rodgers, A., et al., eds., Comparative Quantification of Health Risks: Global and Regional Burden of Disease Attributable to Selected Major Risk Factors, World Health Organization, Geneva, 497-596. | metaanalysis  (not specified) |
| Konnopka, A. / Lehnert, T. | Calle EE, Rodriguez C, Walker-Thurmond K, Thun MJ. Overweight, obesity, and mortality from cancer in a prospectively studied cohort of U.S. adults. N Engl J Med. 2003 Apr 24;348(17):1625-38. doi: 10.1056/NEJMoa021423. PMID: 12711737. | cohort study |
|  | Must A, Spadano J, Coakley EH, Field AE, Colditz G, Dietz WH. The disease burden associated with overweight and obesity. JAMA. 1999 Oct 27;282(16):1523-9. doi: 10.1001/jama.282.16.1523. PMID: 10546691. | cross sectional study |
| Kristina, S. A. | Guh DP, Zhang W, Bansback N, Amarsi Z, Birmingham CL, Anis AH. The incidence of co-morbidities related to obesity and overweight: a systematic review and meta-analysis. BMC Public Health. 2009 Mar 25;9:88. doi: 10.1186/1471-2458-9-88. PMID: 19320986; PMCID: PMC2667420. | systematic review and metaanalysis of cohort studies |
| Luo, W. | Flegal KM, Graubard BI, Williamson DF, Gail MH. Excess deaths associated with underweight, overweight, and obesity. JAMA. 2005 Apr 20;293(15):1861-7. doi: 10.1001/jama.293.15.1861. PMID: 15840860. | cohort study |
| Martin-Ramiro, J. J. | Prospective Studies Collaboration; Whitlock G, Lewington S, Sherliker P, Clarke R, Emberson J, Halsey J, Qizilbash N, Collins R, Peto R. Body-mass index and cause-specific mortality in 900 000 adults: collaborative analyses of 57 prospective studies. Lancet. 2009 Mar 28;373(9669):1083-96. doi: 10.1016/S0140-6736(09)60318-4. Epub 2009 Mar 18. PMID: 19299006; PMCID: PMC2662372. | metaanalysis  of prospective studies |
| Ni Mhurchu,C. | James, W.P.T., Jackson-Leach, R. and NiMhurchu, C. (2004) Overweight and obesity (high body mass index). In: Ezzati, M., Lopez, A., Rodgers, A., et al., eds., Comparative Quantification of Health Risks: Global and Regional Burden of Disease Attributable to Selected Major Risk Factors, World Health Organization, Geneva, 497-596. | metaanalysis  (not specified) |
|  | Ni Mhurchu C, Rodgers A, Pan WH, Gu DF, Woodward M; Asia Pacific Cohort Studies Collaboration. Body mass index and cardiovascular disease in the Asia-Pacific Region: an overview of 33 cohorts involving 310 000 participants. Int J Epidemiol. 2004 Aug;33(4):751-8. doi: 10.1093/ije/dyh163. Epub 2004 Apr 22. PMID: 15105409. | metaanalysis  of cohort studies |
| Riquelme, R. | Global BMI Mortality Collaboration, Di Angelantonio E, Bhupathiraju ShN, Wormser D, Gao P, Kaptoge S, Berrington de Gonzalez A, Cairns BJ, Huxley R, Jackson ChL, Joshy G, Lewington S, Manson JE, Murphy N, Patel AV, Samet JM, Woodward M, Zheng W, Zhou M, Bansal N, Barricarte A, Carter B, Cerhan JR, Smith GD, Fang X, Franco OH, Green J, Halsey J, Hildebrand JS, Jung KJ, Korda RJ, McLerran DF, Moore SC, O'Keeffe LM, Paige E, Ramond A, Reeves GK, Rolland B, Sacerdote C, Sattar N, Sofianopoulou E, Stevens J, Thun M, Ueshima H, Yang L, Yun YD, Willeit P, Banks E, Beral V, Chen Zh, Gapstur SM, Gunter MJ, Hartge P, Jee SH, Lam TH, Peto R, Potter JD, Willett WC, Thompson SG, Danesh J, Hu FB. Body-mass index and all-cause mortality: individual-participant-data meta-analysis of 239 prospective studies in four continents. Lancet. 2016 Aug 20;388(10046):776-86. doi: 10.1016/S0140-6736(16)30175-1. Epub 2016 Jul 13. PMID: 27423262; PMCID: PMC4995441. | metaanalysis of prospective studies |
| Vidra, N. - 1 | Flegal KM, Kit BK, Orpana H, Graubard BI. Association of all-cause mortality with overweight and obesity using standard body mass index categories: a systematic review and meta-analysis. JAMA. 2013 Jan 2;309(1):71-82. doi: 10.1001/jama.2012.113905. PMID: 23280227; PMCID: PMC4855514. | metaanalysis  of cohort studies |
|  | Wang Z. Age-dependent decline of association between obesity and mortality: a systematic review and meta-analysis. Obes Res Clin Pract. 2015 Jan-Feb;9(1):1-11. doi: 10.1016/j.orcp.2014.01.006. Epub 2014 Mar 6. PMID: 25660170. | systematic review  and metaanalysis  of cohort studies |
|  | Lobstein, T.; Leach, R.J. Workpackage 7: Overweight and Obesity Report on Data Collection for Overweight and Obesity Prevalence and Related Relative Risks. 2010. Available online: https://www.dynamo-hia.eu/sites/default/files/2018-04/BMI_WP7-datareport_20100317.pdf | NS |
|  | Danaei G, Ding EL, Mozaffarian D, Taylor B, Rehm J, Murray CJ, Ezzati M. The preventable causes of death in the United States: comparative risk assessment of dietary, lifestyle, and metabolic risk factors. PLoS Med. 2009 Apr 28;6(4):e1000058. doi: 10.1371/journal.pmed.1000058. Epub 2009 Apr 28. Erratum in: PLoS Med. 2011 Jan;8(1). doi: 10.1371/annotation/0ef47acd-9dcc-4296-a897-872d182cde57. PMID: 19399161; PMCID: PMC2667673. | analysis  of cohorts |
| Vidra, N. - 2 | Flegal KM, Kit BK, Orpana H, Graubard BI. Association of all-cause mortality with overweight and obesity using standard body mass index categories: a systematic review and meta-analysis. JAMA. 2013 Jan 2;309(1):71-82. doi: 10.1001/jama.2012.113905. PMID: 23280227; PMCID: PMC4855514. | metaanalysis  of cohort studies |
| Wen, C. P. | Ho MS (1993) A Long-term Follow-up Study of Chronic Diseases among Civil Servants and Teachers in Taiwan. The Project Report. Taiwan: Institute of Biomedical Science, Academia Sinica. | NS |
|  | Wen CP, Tsai SP, Chen CJ & Cheng TY (2004) The mortality risks of smokers in Taiwan. Part I: cause-specific mortality. Prev Med 39, 528–535. | analysis of cohorts |
|  | Wen CP, Cheng TY, Tsai SP, Hsu HL, Wang SL. Increased mortality risks of pre-diabetes (impaired fasting glucose) in Taiwan. Diabetes Care. 2005 Nov;28(11):2756-61. doi: 10.2337/diacare.28.11.2756. PMID: 16249552. | analysis of cohorts |
| Xu, L. S. | Schmid D, Ricci C, Behrens G, Leitzmann MF. Adiposity and risk of thyroid cancer: a systematic review and meta-analysis. Obes Rev. 2015 Dec;16(12):1042-54. doi: 10.1111/obr.12321. Epub 2015 Sep 14. PMID: 26365757. | metaanalysis  of case-control and cohort studies |
|  | Niedermaier T, Behrens G, Schmid D, Schlecht I, Fischer B, Leitzmann MF. Body mass index, physical activity, and risk of adult meningioma and glioma: A meta-analysis. Neurology. 2015 Oct 13;85(15):1342-50. doi: 10.1212/WNL.0000000000002020. Epub 2015 Sep 16. PMID: 26377253. | metaanalysis  of case-control and cohort studies |
|  | Zhang Y, Liu H, Yang S, Zhang J, Qian L, Chen X. Overweight, obesity and endometrial cancer risk: results from a systematic review and meta-analysis. Int J Biol Markers. 2014 Mar 24;29(1):e21-9. doi: 10.5301/jbm.5000047. PMID: 24170556. | meta-analysis  of case-control and cohort studies |
|  | Jiao L, Berrington de Gonzalez A, Hartge P, Pfeiffer RM, Park Y, Freedman DM, Gail MH, Alavanja MC, Albanes D, Beane Freeman LE, Chow WH, Huang WY, Hayes RB, Hoppin JA, Ji BT, Leitzmann MF, Linet MS, Meinhold CL, Schairer C, Schatzkin A, Virtamo J, Weinstein SJ, Zheng W, Stolzenberg-Solomon RZ. Body mass index, effect modifiers, and risk of pancreatic cancer: a pooled study of seven prospective cohorts. Cancer Causes Control. 2010 Aug;21(8):1305-14. doi: 10.1007/s10552-010-9558-x. Epub 2010 Apr 10. PMID: 20383573; PMCID: PMC2904431. | pooled analysis  of prospective cohorts |
|  | Yang C, Lu Y, Xia H, Liu H, Pan D, Yang X, Sun G. Excess Body Weight and the Risk of Liver Cancer: Systematic Review and a Meta-Analysis of Cohort Studies. Nutr Cancer. 2020;72(7):1085-1097. doi: 10.1080/01635581.2019.1664602. Epub 2019 Sep 23. PMID: 31544511. | metaanalysis  of cohort studies |
|  | Tan W, Gao M, Liu N, Zhang G, Xu T, Cui W. Body Mass Index and Risk of Gallbladder Cancer: Systematic Review and Meta-Analysis of Observational Studies. Nutrients. 2015 Sep 25;7(10):8321-34. doi: 10.3390/nu7105387. PMID: 26426043; PMCID: PMC4632410. | meta-analysis  of case-control and cohort studies |
|  | P. Yang, Y. Zhou, B. Chen, H.-W. Wan, G.-Q. Jia, H.-L. Bai, X.-T. Wu, Overweight, obesity and gastric cancer risk: results from a meta-analysis of cohort studies, Eur. J. Cancer 45 (2009) 2867–2873, https://doi.org/10.1016/j.ejca.2009.04.019. | metaanalysis  of cohort studies |
|  | Chen X, Lu W, Zheng W, Gu K, Chen Z, Zheng Y, Shu XO. Obesity and weight change in relation to breast cancer survival. Breast Cancer Res Treat. 2010 Aug;122(3):823-33. doi: 10.1007/s10549-009-0708-3. Epub 2010 Jan 8. PMID: 20058068; PMCID: PMC3777404. | analysis of cohorts |
|  | G.E. Nam, K.H. Cho, K. Han, C.M. Kim, B. Han, S.J. Cho, S.J. Jung, Y. Kwon, Y. H. Kim, D.H. Kim, S.M. Kim, Y.S. Choi, Y.K. Roh, Y.G. Park, Obesity, abdominal obesity and subsequent risk of kidney cancer: a cohort study of 23.3 million East Asians, Br. J. Cancer 121 (2019) 271–277, https://doi.org/10.1038/s41416-019-0500-z. | analysis of cohorts |
|  | B.M. Birmann, G. Andreotti, A.J.D. Roos, N.J. Camp, B.C.H. Chiu, J.J. Spinelli, N. Becker, V. Benhaim-Luzon, P. Bhatti, P. Boffetta, P. Brennan, E.E. Brown, P. Cocco, L. Costas, W. Cozen, Young adult and usual adult body mass index and multiple myeloma risk: a pooled analysis in the International Multiple Myeloma Consortium (IMMC), (2018) 20 | pooled analysis of prospective cohorts |
|  | C.L. Parr, G.D. Batty, T.H. Lam, F. Barzi, X. Fang, S.C. Ho, S.H. Jee, A. Ansary- Moghaddam, K. Jamrozik, H. Ueshima, M. Woodward, R.R. Huxley, Body-mass index and cancer mortality in the Asia-Pacific Cohort Studies Collaboration: pooled analyses of 424 519 participants, Lancet Oncol. 11 (2010) 741–752, https://doi. org/10.1016/S1470-2045(10)70141-8. | pooled analysis of prospective cohorts |
